# 40 years after the discovery of *Helicobacter Pylori*: Performing optimized “subtraction” for clinical eradication

**DOI:** 10.1002/imo2.70017

**Published:** 2025-04-07

**Authors:** Yi Hu, Ren‐Chun Du, Yong‐Kang Lai, Yu‐Xin Hu, Yu‐Chen Zhu, Yan‐An Zhou, Chun‐Xi Shu, Bo Zhou, Li‐Xiang Ling, Xu Shu, Yong Xie, James Y. W. Lau, Yin Zhu, David Y. Graham, Nong‐Hua Lu

**Affiliations:** ^1^ Jiangxi Provincial Key Laboratory of Digestive Diseases, Department of Gastroenterology, The First Affiliated Hospital, Jiangxi Medical College Nanchang University Nanchang Jiangxi China; ^2^ Department of Surgery at the Sir YK Pao Centre for Cancer The Chinese University of Hong Kong Hong Kong China; ^3^ Huankui Academy Nanchang University Nanchang Jiangxi Province China; ^4^ Department of Gastroenterology Changhai Hospital, Naval Medical University Shanghai China; ^5^ Department of Medicine Michael E. DeBakey VA Medical Center, and Baylor College of Medicine Houston Texas USA

## Abstract

It has been 40 years since the first discovery of the important human pathogen, *Helicobacter pylori* (*H. pylori*). Eradication of the infection continues to pose significant challenges in part because of the global increase in antibiotic resistance. Generally, the “addition” strategy has failed to deliver the desired results for more than a short period and often contributed to the increase in the prevalence of resistant strains. As such, optimization of each component of the treatment strategy has become an increasingly critical focus of research. In this study, we summarized the evolution of *H. pylori* eradication regimens and the emergence of promising regimens (e.g., high‐dose pronto pump inhibitor dual therapy and potassium‐competitive acid blocker dual therapy) in clinical practice. Moreover, we conducted an updated meta‐analysis incorporating a total of 29 clinical studies regarding potassium‐competitive acid blocker dual therapy. Additionally, we also conducted a network meta‐analysis regarding the evaluation of first‐line regimens for eradicating *H. pylori* among patients with penicillin allergy. We concluded that the strategy for the first‐line treatment of *H. pylori* infection needs to shift from the traditional “addition” approach to an optimized “subtraction” approach in this era of increasing antibiotic resistance. Dual therapies, particularly those involving vonoprazan and low‐dose amoxicillin, have demonstrated both satisfactory eradication rates and patient adherence.

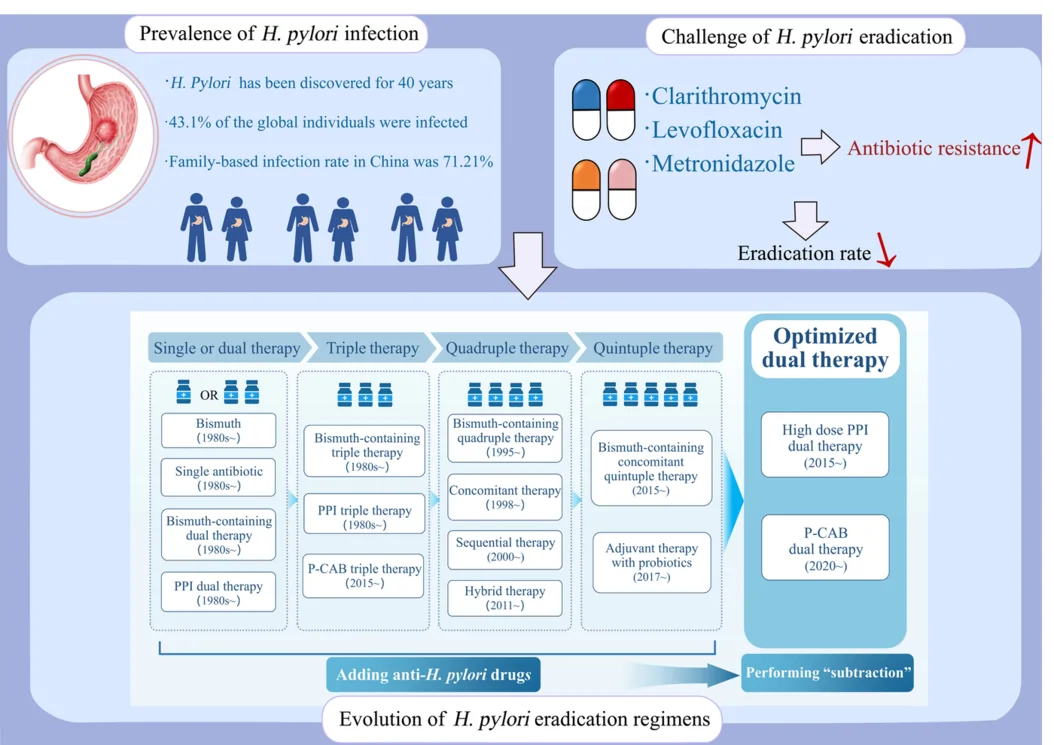

## ETHICS STATEMENT

No animals or humans were involved in this study.

To the editor,

It has been 40 years since the first discovery of the important human pathogen, *Helicobacter pylori* (*H. pylori*). Eradication of the infection continues to pose significant challenges in part because of the global increase in antibiotic resistance [1]. This infectious disease has been largely managed by gastroenterologists rather than specialists in infectious disease and one response to reduced cure rates has typically been to increase the dosage or variety of antibiotics and antisecretory drugs used as well as adding bismuth. Generally, this “addition” strategy has failed to deliver the desired results for more than a short period and often contributed to the increase in the prevalence of resistant strains. As such, optimization of each component of the treatment strategy has become an increasingly critical focus of research.

### Evolution of *H. pylori* regimens

**FIGURE 1:**
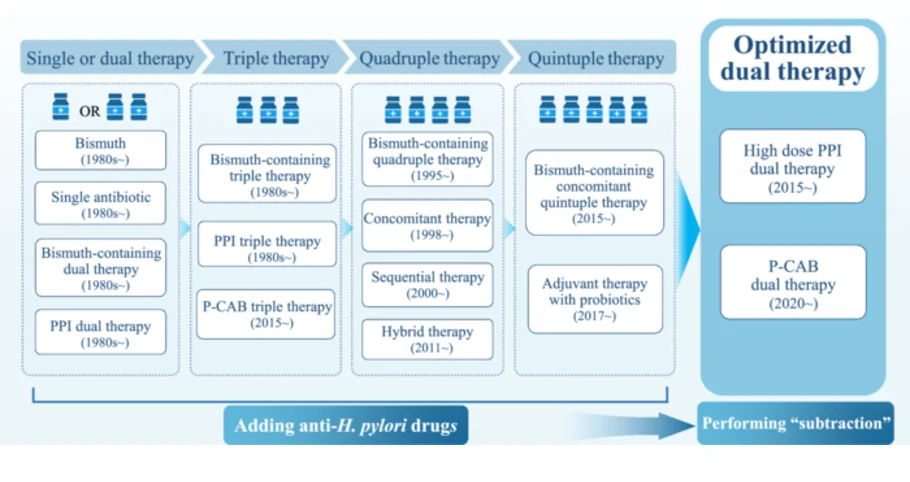
Evolution of *H. pylori* eradication therapies. PPI, pronto pump inhibitor; P‐CAB, potassium‐competitive acid blocker.

*H. pylori* eradication strategies have continued to evolve, as illustrated in Figure 1. The earliest effective treatment strategies for *H. pylori* infection employed bismuth, metronidazole, and tetracycline. A Proton Pump Inhibitor (PPI) was subsequently added to overcome metronidazole resistance. Clarithromycin and omeprazole (20 mg, once or twice daily) were attempted but failed because of the development of resistance during therapy. The addition of amoxicillin (1 g, twice daily) to clarithromycin was introduced in the late 1980s to prevent the emergence of resistance during therapy [2].

The problem of resistance to clarithromycin resurfaced rapidly, resulting in the addition of new triple and quadruple therapies such as sequential therapy, hybrid therapy, and concomitant therapy. However, these regimens suffered from inconvenient drug administration, causing problems with patient adherence. In addition, they often contained unneeded antibiotics, which conflicted with the principles of antibiotic stewardship. Additionally, the antibiotics that local infections were susceptible to were often not universally available [3]. Moreover, efforts to improve the efficacy of regimens and decrease the rate of side effects also resulted in an increased dysbiosis of gut microbiota. Although probiotics were incorporated, their addition generally failed to deliver the anticipated benefits in terms of improved efficacy. However, probiotics often decrease the rate of side effects and improve the dysbiosis of gut microbiota [4].

All proposed novel regimens should consider the availability of the regimen, the technical details of the application of the regimen, and the impact of the regimen on the host (i.e., secondary resistance of antibiotics and dysbiosis of gut microbiota). We conducted a pilot study to evaluate the 7‐day bismuth‐containing concomitant quintuple regimen (bismuth plus clarithromycin, amoxicillin, metronidazole, and PPI) for *H. pylori* eradication. After 70 patients were included, it became clear that the efficacy was unacceptable (<80%). In addition, the therapy was associated with a high rate of side effects (>30%) [5], and despite the regimen incorporating up to five drugs, resistance rates continued to rise, leading to a decline in cure rates. As such, using the “addition” strategy for the purpose of increasing efficacy has proven not to be an effective strategy.

Bismuth‐containing quadruple therapy is widely recommended by international and Chinese consensus reports. Additionally, Maastricht VI/Florence consensus report stated that high‐dose dual therapy (HDDT) might be recommended after failure of first‐line treatment of *H. pylori* [6]. However, HDDT is recommended as first‐line or second‐line treatment of *H. pylori* by Chinese consensus report, the difference of recommendation might be explained by the fact that majority of dual therapy for eradicating *H. pylori* was completed in China [7]. Potassium‐competitive acid blocker (P‐CAB) is recommended to be used in *H. pylori* regimens by international and Chinese consensus reports because of its strong, quick and long‐lasting inhibition of gastric acids.

Effective eradication of *H. pylori* involves several critical factors. First, all antimicrobial treatments should be guided by susceptibility testing and optimized to reliably achieve high cure rates (≥95%). In most regions, bismuth‐containing quadruple therapy can be used successfully as an empirical treatment option. However, some popular empirical therapies (e.g., sequential, concomitant, or hybrid therapies) often result in the inclusion of at least one unnecessary antibiotic that does not improve treatment efficacy and contributes to the global rise of antibiotic resistance and an eventual significant decline in cure rates. Therefore, effective *H. pylori* regimens should exclude unnecessary antibiotics and therapies containing agents with no effect on outcomes should be avoided. The empirical use of metronidazole should be restricted to bismuth‐containing quadruple therapy. Triple therapies involving clarithromycin, metronidazole, or levofloxacin should only be administered as susceptibility‐based therapies unless local resistance rates are proven to be low and regional efficacy is regularly validated [8]. In addition, minimizing adverse effects and ensuring patient adherence are key to the successful use of otherwise effective regimens. Moreover, the cost‐effectiveness and accessibility of antibiotics need to be considered. Amoxicillin and tetracycline are widely available and affordable in most regions and are associated with low rates of resistance [9]. As such, the unique benefits of amoxicillin and tetracycline highlight their promising role in *H. pylori* eradication regimens.

### Emergence of promising regimens

As with other infectious diseases, single antibiotic regimens are most desired. Treatment of intragastric infections is complicated by the presence of acid and by the rapid emptying of gastric contents. HDDT combining amoxicillin with an antisecretory agent has shown that effective therapy is possible with few adverse reactions and low treatment costs. The proof of principle initially came from clinical trials of amoxicillin and omeprazole [10]. However, the approach proved to be effective only in areas where atrophic gastritis (low acid secretion) was present. Later, Yang et al. [11] revisited the problem in 2015 using increased antisecretory therapy. They demonstrated satisfactory eradication rates of 95.3% and 96.6% by intent to treat and per protocol analysis. The introduction of vonoprazan, the currently most effective antisecretory agent, proved that high cure rates were possible in some regions of China and Japan. Ideally, cure rates should be reliably above 90% and preferably 95% or higher in adherent patients. A number of meta‐analyses have been performed using vonoprazan or high‐dose potency PPI (e.g., 40 mg of esomeprazole or rabeprazole b.i.d.) and high‐dose of amoxicillin such as 1 g t.i.d. or 750 mg q.i.d [12]. In Japan, similar high cure rates have been achieved with low‐dose amoxicillin. In high populations with larger individuals with increased Body Mass Index, reliable achievement of high cure rates has not been reliably obtained [13].

Our previous meta‐analysis demonstrated that compared with vonoprazan triple therapy, vonoprazan and amoxicillin (VA) dual therapy achieved high eradication rates with a simplified treatment process and improved patient adherence (i.e., the addition of clarithromycin to VA dual therapy did not enhance efficacy for most patients and resulted in a prescription for unneeded antibiotics) [14]. Considering its high efficacy, low side effects and good patient adherence, VA dual therapy has emerged as a promising option for the treatment of *H. pylori* infection. As more clinical trials have been published, we conducted an updated meta‐analysis incorporating a total of 29 clinical studies regarding P‐CAB dual therapy, which allowed us to evaluate the eradication rates across different treatment durations and drug combinations. As shown in Figure S1, the overall eradication rate for P‐CAB dual therapy was 91.5%. Specifically, the pooled eradication rates for VA dual therapy were 82.4% for the 7‐day regimen, 90.7% for the 10‐day regimen, and 93.9% for the 14‐day regimen.

These data illustrate the need for studies using a factorial design to optimize the dosages of the P‐CAB and the antibiotic (e.g., amoxicillin, tetracycline or possibly rifabutin) dual therapies so that they each reliably achieve cure rates of ≥95% in the local population. A preliminary study in Australia suggested that vonoprazan 20 mg t.i.d. might be sufficient to reliably achieve an appropriate intragastric milieu in Western patients [15].

Numerous studies have reported that both low‐dose and high‐dose amoxicillin dual therapies are effective. We also conducted a nationwide, multi‐center study specifically assessing the efficacy of vonoprazan combined with low or high‐dose amoxicillin that included 504 patients from different regions across the country. The results suggested that no significant difference was observed in efficacy between the low‐dose (2 grams per day) and high‐dose (3 grams per day) amoxicillin groups (88.8% vs. 92.4%). Moreover, no patients were resistant to amoxicillin or tetracycline, and the impact on gut microbiota and resistance genes was relatively minimal [16]. These findings further support the concept that a simplified dual therapy, particularly with low‐dose amoxicillin, can effectively improve eradication rates while minimize adverse effects and showed a temporary impact for the emergence of antibiotics‐related resistance genes.

Moreover, 14‐day regimen of vonoprazan combined with tetracycline conducted by Gao et al. [17] surprisingly reached an eradication rate of 95.1% among patients with penicillin allergy. These studies offered a novel option for P‐CAB dual therapy that included an alternative antibiotic, tetracycline. We also conducted a network meta‐analysis regarding the evaluation of first‐line regimens for eradicating *H. pylori* among patients with penicillin allergy. A total of 5 studies involving 931 patients were included. As shown in Figure S2, vonoprazan dual and triple therapy was non‐inferior to PPI‐triple therapy and bismuth‐containing quadruple therapy in each study. Moreover, bismuth‐containing quadruple therapy ranked first in efficacy, followed by vonoprazan triple therapy, VA therapy and PPIs triple therapy. However, bismuth‐containing quadruple therapy showed the highest rate of adverse events rate, followed by PPI triple therapy, vonoprazan triple therapy and vonoprazan dual therapy, suggesting that vonoprazan‐tetracycline dual therapy may have the highest safety and compliance in patients with penicillin allergy (Table S1).

### Effects of VA dual therapy on gut microbiota

The widespread use of antibiotics to eradicate *H. pylori* as a preventive measure against gastric cancer and peptic ulcers has raised several concerns, including antibiotic resistance and disturbances in the gut microbiota. Short‐term antibiotic treatment for *H. pylori* eradication can significantly disturb the gastrointestinal microbiota, leading to imbalances in oral and gut microbial communities after the completion of treatment [18]. The extent and severity of microbiota disturbances following *H. pylori* eradication vary depending on the regimens, partly due to differences in the dosage, frequency, and duration of PPI, P‐CAB, and antibiotics. Numerous studies have demonstrated significant disruptions in the diversity and composition of the gut microbiota after *H. pylori* eradication, particularly with triple therapy and bismuth‐containing quadruple therapy [19]. Generally, the alpha diversity and abundance of the gut microbiota decrease shortly after eradication therapy and gradually recover over time. Concurrently, the diversity of antibiotic‐related resistance genes increases but tends to subside within 6 months [20]. In our previously mentioned recent multi‐center study to evaluate the efficacy of vonoprazan with low‐dose or high‐dose amoxicillin for eradicating *H. pylori*, we found that the Alpha diversity of gut microbiota was decreased after treatment in both groups, but recovered to pretreatment levels at week 8‐10. Additionally, the abundance of beta‐lactam‐related resistance genes increased after treatment but was restored to pretreatment for low‐dose VA dual therapy, not high‐dose VA dual therapy [16].

**FIGURE 2:**
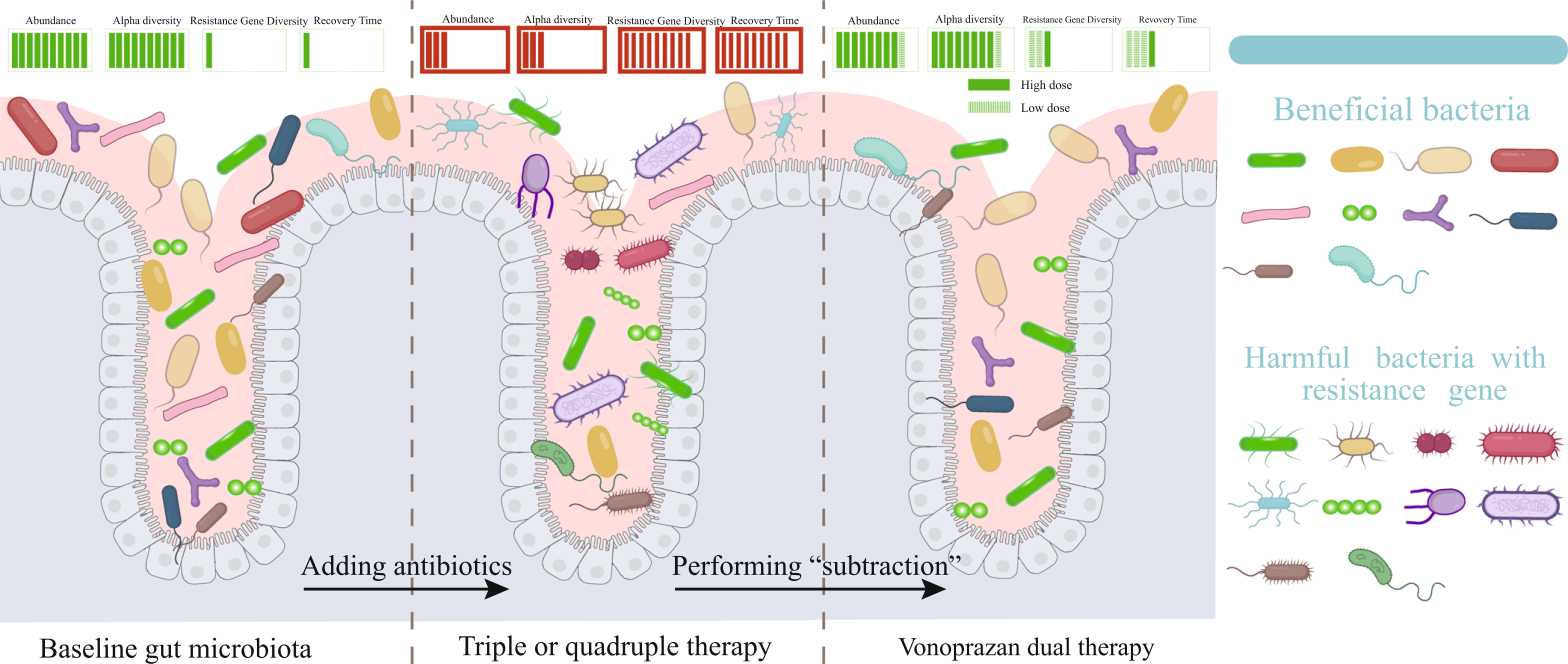
The impacts of different *H. pylori* treatment regimens on gut microbiota.

The impacts of different *H. pylori* regimens on gut microbiota are shown in Figure 2. Compared with triple and quadruple therapy, dual therapy, especially low‐dose VA dual therapy, which involves fewer drugs and leads to fewer side effects and a reduced impact on gut microbiota and resistance genes, making it more suitable for wide application in clinical practice.

### Evaluation and future perspectives

The strategy for the first‐line treatment of *H. pylori* infection needs to shift from the traditional “addition” approach to an optimized “subtraction” approach in this era of increasing antibiotic resistance. Simplifying treatment regimens, improving patient compliance, reducing antibiotic usage, and optimizing therapeutic strategies represent the future direction for *H. pylori* eradication. Dual therapies, particularly those involving vonoprazan and low‐dose amoxicillin, have demonstrated both satisfactory eradication rates and patient adherence. Additionally, novel antibiotic combinations warrant further exploration, possibly including the potential efficacy of next‐generation tetracyclines such as minocycline and doxycycline.

## AUTHOR CONTRIBUTIONS

**Yi Hu**: Conceptualization; investigation; funding acquisition; writing—original draft; writing—review and editing; methodology; validation; supervision; formal analysis; visualization. **Ren‐Chun Du**: Data curation; software; conceptualization; methodology; writing—original draft; writing—review and editing; validation; formal analysis; visualization. **Yong‐Kang Lai**: Conceptualization; methodology; software; investigation; validation. **Yu‐Xin Hu**: Conceptualization; methodology; software; investigation; validation. **Yu‐Chen Zhu**: Conceptualization; methodology; software; validation; investigation. **Yan‐An Zhou**: Conceptualization; investigation; data curation. **Chun‐Xi Shu**: Conceptualization; investigation; data curation. **Bo Zhou**: Conceptualization; investigation; data curation. **Li‐Xiang Ling**: Conceptualization; investigation; data curation. **Xu Shu**: Writing—review and editing; project administration; resources. **Yong Xie**: Resources; project administration; writing—review and editing. **James Y. W. Lau**: Writing—review and editing; resources; supervision. **Yin Zhu**: Writing—review and editing; methodology; software. **David Y. Graham**: Writing—review and editing; conceptualization; investigation; methodology; validation; software; formal analysis; supervision. **Nong‐Hua Lu**: Conceptualization; investigation; validation; methodology; writing—review and editing; software; formal analysis; supervision.

## CONFLICT OF INTEREST STATEMENT

The authors declare no conflicts of interest.

## Supporting information


**Figure S1:** The pooled eradication rate of P‐CAB dual therapy.
**Figure S2:** Forest plots for the comparison of different *H. pylori* eradication regimens for patients allergic to penicillin in efficacy. V, vonoprazan; P, proton pump inhibitor; T, tetracycline; C, clarithromycin; M, metronidazole; S, sitafloxacin; B, bismuth; Cef, cefuroxime; Min, minocycline.

**Table S1:** Surface Under the Cumulative Ranking Curve‐based efficacy ranking league matrix showing the comparative efficacies and adverse events rates of the regimens.

## Data Availability

The data that support the findings of this study are available in the supporting information. Supplementary materials (figures and tables) may be found in the online DOI or iMetaOmics http://www.imeta.science/imetaomics/.
