# Supplementary material for: 40 years after the discovery of Helicobacter Pylori: Performing optimized “subtraction” for clinical eradication
Source: IMetaOmics. 2025 Apr 7;2(2):e70017. doi: 10.1002/imo2.70017 (PMC12806322; doi:10.1002/imo2.70017)
Supplement: Supplementary file 1 — Figure S1: The pooled eradication rate of P‐CAB dual therapy. Figure S2: Forest plots for the comparison of different H. pylori eradication regimens for patients allergic to penicillin in efficacy. V, vonoprazan; P, proton pump inhibitor; T, tetracycline; C, clarithromycin; M, metronidazole; S, sitafloxacin; B, bismuth; Cef, cefuroxime; Min, minocycline. [file IMO2-2-e70017-s002.docx]

**Supporting Information to:**

**40 Years After the Discovery of *Helicobacter Pylori*: Performing Optimized “Subtraction” for Clinical Eradication**

Yi Hu ^1,2#^, Ren-Chun Du ^1,3#^, Yong-Kang Lai ^4^, Yu-Xin Hu ^1^, Yu-Chen Zhu ^1,3^, Yan-An Zhou ^1^, Chun-Xi Shu ^1^, Bo Zhou ^1^, Li-Xiang Ling ^1^, Xu Shu ^1^, Yong Xie ^1^, James YW Lau ^2^, Yin Zhu ^1*^, David Y. Graham ^5*^, Nong-Hua Lu ^1*^

^#^ These authors contributed equally as first authors.

^*^Contributed equally as corresponding authors.

1. Jiangxi Provincial Key Laboratory of Digestive Diseases, Department of Gastroenterology, The First Affiliated Hospital, Jiangxi Medical College, Nanchang University, Nanchang 330006, Jiangxi, China
2. Department of Surgery at the Sir YK Pao Centre for Cancer, The Chinese University of Hong Kong, Hong Kong 999077, China.
3. Huankui Academy, Nanchang University, Nanchang 330031, Jiangxi Province, China.
4. Department of Gastroenterology, Changhai Hospital, Naval Medical University, Shanghai 200433, China.
5. Department of Medicine, Michael E. DeBakey VA Medical Center, and Baylor College of Medicine, Houston, TX, 77030, USA.

Correspondence should be addressed to:

Lu Nong-Hua; Department of Gastroenterology, The First Affiliated Hospital of Nanchang University, 17 Yong Waizheng Street, Donghu District, Nanchang 330006, Jiangxi Province, China. lunonghua@ncu.edu.cn.

David Y. Graham; Michael E. DeBakey Veterans Affairs Medical Center, RM 3A-318B (111D), 2002 Holcombe Boulevard, Houston, TX 77030 USA. [dgraham@bcm.edu](mailto:dgraham@bcm.edu).

Zhu Yin; Department of Gastroenterology, The First Affiliated Hospital of Nanchang University, 17 Yong Waizheng Street, Donghu District, Nanchang 330006, Jiangxi Province, China. ndyfy01977@ncu.edu.cn.

**Supplementary figure**


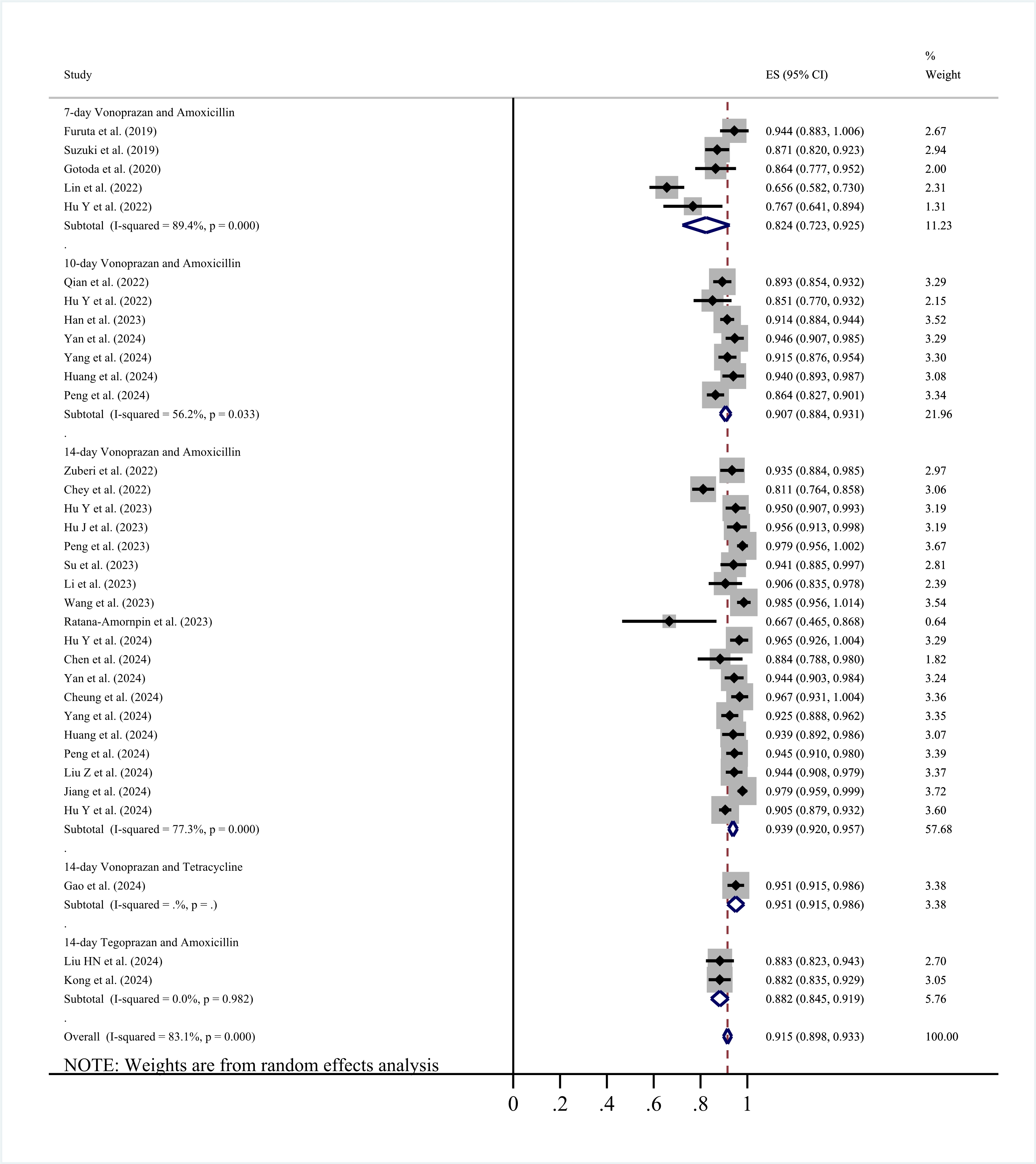


**Figure S1** The pooled eradication rate of P-CAB dual therapy.


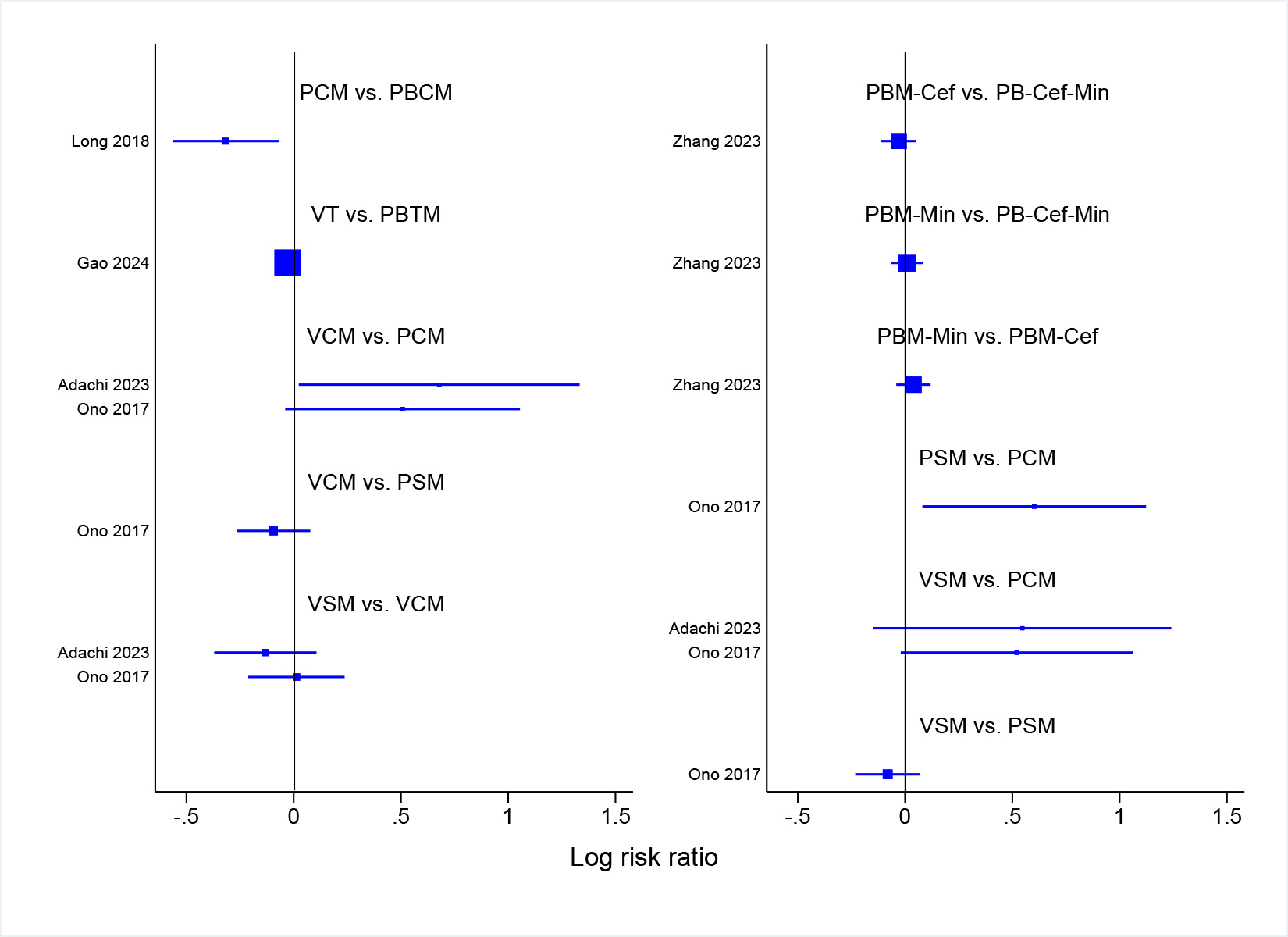


**Figure S2** Forest plots for the comparison of different *H. pylori* eradication regimens

for patients allergic to penicillin in efficacy. V, vonoprazan; P, proton pump inhibitor;

T, tetracycline; C, clarithromycin; M, metronidazole; S, sitafloxacin; B, bismuth; Cef,

cefuroxime; Min, minocycline.

**Reference:**

1. Furuta, T., M. Yamade, T. Kagami, T. Uotani, T. Suzuki, T. Higuchi, S. Tani, et al. 2020. “Dual Therapy with Vonoprazan and Amoxicillin Is as Effective as Triple Therapy with Vonoprazan, Amoxicillin and Clarithromycin for Eradication of Helicobacter pylori.” *Digestion* 101: 743-751. <https://doi.org/10.1159/000502287>

2. Suzuki, S., T. Gotoda, C. Kusano, H. Ikehara, R. Ichijima, M. Ohyauchi, H. Ito, et al. 2020. “Seven-day vonoprazan and low-dose amoxicillin dual therapy as first-line Helicobacter pylori treatment: a multicentre randomised trial in Japan.” *Gut* 69: 1019-1026. <https://doi.org/10.1136/gutjnl-2019-319954>

3. Gotoda, T., C. Kusano, S. Suzuki, T. Horii, R. Ichijima, H. Ikehara. 2020. “Clinical impact of vonoprazan-based dual therapy with amoxicillin for H. pylori infection in a treatment-naïve cohort of junior high school students in Japan.” *J Gastroenterol* 55: 969-976. <https://doi.org/10.1007/s00535-020-01709-4>

4. Qian, H. S., W. J. Li, Y. N. Dang, L. R. Li, X. B. Xu, L. Yuan, W. F. Zhang, et al. 2023. “Ten-Day Vonoprazan-Amoxicillin Dual Therapy as a First-Line Treatment of Helicobacter pylori Infection Compared With Bismuth-Containing Quadruple Therapy.” *Am J Gastroenterol* 118: 627-634. <https://doi.org/10.14309/ajg.0000000000002086>

5. Zuberi, B. F., F. S. Ali, T. Rasheed, N. Bader, S. M. Hussain, A. Saleem. 2022. “Comparison of Vonoprazan and Amoxicillin Dual Therapy with Standard Triple Therapy with Proton Pump Inhibitor for Helicobacter Pylori eradication: A Randomized Control Trial.” *Pak J Med Sci* 38: 965-969. <https://doi.org/10.12669/pjms.38.4.5436>

6. Chey, W. D., F. Mégraud, L. Laine, L. J. López, B. J. Hunt, C. W. Howden. 2022. “Vonoprazan Triple and Dual Therapy for Helicobacter pylori Infection in the United States and Europe: Randomized Clinical Trial.” *Gastroenterology* 163: 608-619. <https://doi.org/10.1053/j.gastro.2022.05.055>

7. Lin, Y., H. Xu, J. Yun, X. Yu, Y. Shi, D. Zhang. 2022. “The efficacy of vonoprazan combined with different dose amoxicillin on eradication of Helicobacter pylori: an open, multicenter, randomized clinical study.” *Ann Transl Med* 10: 987. <https://doi.org/10.21037/atm-22-4133>

8. Hu, Y., X. Xu, Y. B. Ouyang, C. He, N. S. Li, C. Xie, C. Peng, et al. 2022. “Optimization of vonoprazan-amoxicillin dual therapy for eradicating Helicobacter pyloriinfection in China: A prospective, randomized clinical pilot study.” *Helicobacter* 27: e12896. <https://doi.org/10.1111/hel.12896>

9. Hu, Y., X. Xu, X. S. Liu, C. He, Y. B. Ouyang, N. S. Li, C. Xie, et al. 2022. “Fourteen-day vonoprazan and low- or high-dose amoxicillin dual therapy for eradicating Helicobacter pylori infection: A prospective, open-labeled, randomized non-inferiority clinical study.” *Front Immunol* 13: 1049908. <https://doi.org/10.3389/fimmu.2022.1049908>

10. Hu, J., H. Mei, N. Y. Su, W. J. Sun, D. K. Zhang, L. L. Fan, P. He, et al. 2023. “Eradication rates of Helicobacter pylori in treatment-naive patients following 14-day vonoprazan-amoxicillin dual therapy: A multicenter randomized controlled trial in China.” *Helicobacter* 28: e12970. <https://doi.org/10.1111/hel.12970>

11. Peng, X., H. W. Chen, Y. Wan, P. Z. Su, J. Yu, J. J. Liu, Y. Lu, M. Zhang, J. Y. Yao, M. Zhi. 2023. “Combination of vonoprazan and amoxicillin as the first-line Helicobacter pylori eradication therapy: a multicenter, prospective, randomized, parallel-controlled study.” *Clin Exp Med* 23: 4011-4019. <https://doi.org/10.1007/s10238-023-01074-5>

12. Su, N. Y., Q. Shi, H. Mei, J. Hu, Y. X. Liu, H. N. Liu, H. Q. Liu, Y. Guo, X. W. Wang, C. H. Lan. 2023. “Efficacy and safety of vonoprazan-based dual therapy and esomeprazole-based dual therapy in eradicating primary Helicobacter pylori infection: A propensity score matching analysis.” *Helicobacter* 28: e13003. <https://doi.org/10.1111/hel.13003>

13. Li, J., L. Lv, Y. Zhu, Z. Zhou, S. He. 2023. “A Modified 14-Day Dual Therapy with Vonoprazan and Amoxicillin Amplified the Advantages Over Conventional Therapies for Eradication of Helicobacter pylori: A Non-Inferiority Clinical Trial.” *Infect Drug Resist* 16: 5637-5645. <https://doi.org/10.2147/idr.S417711>

14. Wang, X., G. Teng, X. Dong, Y. Dai, W. Wang. 2023. “Efficacy and safety of vonoprazan-amoxicillin dual therapy for Helicobacter pylori first-line treatment: a single-center, randomized, controlled trial.” *Therap Adv Gastroenterol* 16: 17562848231190976. <https://doi.org/10.1177/17562848231190976>

15. Ratana-Amornpin, S., L. Sanglutong, T. Eiamsitrakoon, S. Siramolpiwat, D. Y. Graham, V. Mahachai. 2023. “Pilot studies of vonoprazan-containing Helicobacter pylori eradication therapy suggest Thailand may be more similar to the US than Japan.” *Helicobacter* 28: e13019. <https://doi.org/10.1111/hel.13019>

16. Han, Y. Y., L. Zhou, Y. L. Hu, X. W. Ding, H. Long, F. Liu, M. Xu, et al. 2023. “Comparison of vonoprazan-based with rabeprazole-based dual therapy for treatment-naive patients of Helicobacter pylori infection: a prospective, multi-center, randomized controlled study.” *J Gastroenterol* 58: 1167-1177. <https://doi.org/10.1007/s00535-023-02042-2>

17. Hu, Y., X. H. Huang, B. Zhou, M. L. Liu, Y. F. Liu, T. Yu, P. Sun, et al. 2024. “Vonoprazan and amoxicillin dual therapy for 14 days as the first-line treatment of Helicobacter pylori infection: A non-inferiority, randomized clinical trial.” *Helicobacter* 29: e13045. <https://doi.org/10.1111/hel.13045>

18. Chen, C., D. Zhang, S. Huang, F. Zeng, D. Li, X. Zhang, R. Chen, S. Chen, J. Wang, F. Bai. 2024. “Comparison of vonoprazan dual therapy, quadruple therapy and standard quadruple therapy for Helicobacter pylori infection in Hainan: a single-center, open-label, non-inferiority, randomized controlled trial.” *BMC Gastroenterol* 24: 131. <https://doi.org/10.1186/s12876-024-03225-8>

19. Yan, T. L., J. H. Wang, X. J. He, Y. B. Zhu, L. J. Lu, Y. J. Wang, Z. W. Wang, et al. 2024. “Ten-Day Vonoprazan-Amoxicillin Dual Therapy vs Standard 14-Day Bismuth-Based Quadruple Therapy for First-Line Helicobacter pylori Eradication: A Multicenter Randomized Clinical Trial.” *Am J Gastroenterol* 119: 655-661. <https://doi.org/10.14309/ajg.0000000000002592>

20. Cheung, K. S., T. Lyu, Z. Deng, S. Han, L. Ni, J. Wu, J. T. Tan, et al. 2024. “Vonoprazan Dual or Triple Therapy Versus Bismuth-Quadruple Therapy as First-Line Therapy for Helicobacter pylori Infection: A Three-Arm, Randomized Clinical Trial.” *Helicobacter* 29: e13133. <https://doi.org/10.1111/hel.13133>

21. Yang, F., B. Yu, L. Qin, X. Dai. 2023. “A randomized clinical study on the efficacy of vonoprazan combined with amoxicillin duo regimen for the eradication of Helicobacter pylori.” *Medicine (Baltimore)* 102: e35610. <https://doi.org/10.1097/md.0000000000035610>

22. Huang, X. P., Y. J. Liu, S. W. Lin, Y. F. Shao, F. Qiu, Q. W. Qiu, Z. K. Xu, et al. 2024. “Vonoprazan-amoxicillin dual therapy for Helicobacter pylori eradication in Chinese population: A prospective, multicenter, randomized, two-stage study.” *World J Gastroenterol* 30: 3304-3313. <https://doi.org/10.3748/wjg.v30.i27.3304>

23. Gao, W., J. Liu, X. Wang, J. Li, X. Zhang, H. Ye, J. Li, et al. 2024. “Simplified Helicobacter pylori therapy for patients with penicillin allergy: a randomised controlled trial of vonoprazan-tetracycline dual therapy.” *Gut* 73: 1414-1420. <https://doi.org/10.1136/gutjnl-2024-332640>

24. Peng, X., J. Y. Yao, Y. Q. Ma, G. H. Li, H. W. Chen, Y. Wan, D. S. Liang, M. Zhang, M. Zhi. 2024. “Efficacy and Safety of Vonoprazan-Amoxicillin Dual Regimen With Varying Dose and Duration for Helicobacter pylori Eradication: A Multicenter, Prospective, Randomized Study.” *Clin Gastroenterol Hepatol* 22: 1210-1216. <https://doi.org/10.1016/j.cgh.2024.01.022>

25. Liu, Z., D. Sun, L. Kou, L. Jia, J. Hao, J. Zhou, W. Zheng, F. Gao, X. Chen. 2024. “Vonoprazan-amoxicillin dual therapy with different amoxicillin dosages for treatment-naive patients of Helicobacter pylori infection in China: a prospective, randomized controlled study.” *Eur J Gastroenterol Hepatol* 36: 712-719. <https://doi.org/10.1097/meg.0000000000002760>

26. Jiang, G., M. Luo, P. Zheng, Y. Cong, Y. Feng, F. Zhou. 2024. “Eradication rate and safety of vonoprazan-amoxicillin dual therapy for helicobacter pylori eradication: a randomized controlled trial.” *Scand J Gastroenterol* 59: 1229-1233. <https://doi.org/10.1080/00365521.2024.2407898>

27. Hu, Y., Z. Y. Zhang, F. Wang, K. Zhuang, X. Xu, D. S. Liu, H. Z. Fan, et al. 2025. “Effects of amoxicillin dosage on cure rate, gut microbiota, and antibiotic resistome in vonoprazan and amoxicillin dual therapy for Helicobacter pylori: a multicentre, open-label, non-inferiority randomised controlled trial.” *Lancet Microbe* 6: 100975. <https://doi.org/10.1016/j.lanmic.2024.100975>

28. Liu, H. N., R. Wang, Y. Cao, F. Xian, X. J. Bi, D. J. Wu, B. Wang, X. W. Wang, C. H. Lan. 2024. “Comparison of the Efficacy Between the Dual Therapy of Tegoprazan and the Quadruple Therapy of Tegoprazan: A Randomized Controlled Multicenter Study.” *Clin Transl Gastroenterol* 15: e1. <https://doi.org/10.14309/ctg.0000000000000699>

29. Kong, Q., I. A. Mirza, X. Zhang, X. Song, X. Li, Q. Zhang, L. Xu, et al. 2024. “Fourteen-Day Tegoprazan-Amoxicillin Dual Therapy as the First-Line Treatment of Helicobacter pylori Infection (SHARE2301): A Multicenter, Noninferiority, Randomized Clinical Trial.” *Helicobacter* 29: e13098. <https://doi.org/10.1111/hel.13098>
